# Adaptive Road Crack Detection System by Pavement Classification

**DOI:** 10.3390/s111009628

**Published:** 2011-10-12

**Authors:** Miguel Gavilán, David Balcones, Oscar Marcos, David F. Llorca, Miguel A. Sotelo, Ignacio Parra, Manuel Ocaña, Pedro Aliseda, Pedro Yarza, Alejandro Amírola

**Affiliations:** 1 Computer Engineering Department, Polytechnic School, University of Alcalá, Alcalá de Henares, Madrid 28871, Spain; E-Mails: miguel.gavilan@aut.uah.es (M.G.); david.balcones@aut.uah.es (D.B.); oscar.marcos@aut.uah.es (O.M.); sotelo@aut.uah.es (M.A.S.); ignacio@aut.uah.es (I.P.); mocana@depeca.uah.es (M.O.); 2 Infrastructure Management Division, ACCIONA Engineering, c\Marcelina 3, Madrid 28029, Spain; E-Mails: pedro.aliseda.perezmadrid@acciona.es (P.A.); pedro.yarza.alvarez@acciona.es (P.Y.); alejandro.amirola.sanz@acciona.es (A.A.)

**Keywords:** road distress detection, road surface classification, linear features, multi-class SVM, local binary pattern, gray-level co-occurrence matrix

## Abstract

This paper presents a road distress detection system involving the phases needed to properly deal with fully automatic road distress assessment. A vehicle equipped with line scan cameras, laser illumination and acquisition HW-SW is used to storage the digital images that will be further processed to identify road cracks. Pre-processing is firstly carried out to both smooth the texture and enhance the linear features. Non-crack features detection is then applied to mask areas of the images with joints, sealed cracks and white painting, that usually generate false positive cracking. A seed-based approach is proposed to deal with road crack detection, combining Multiple Directional Non-Minimum Suppression (MDNMS) with a symmetry check. Seeds are linked by computing the paths with the lowest cost that meet the symmetry restrictions. The whole detection process involves the use of several parameters. A correct setting becomes essential to get optimal results without manual intervention. A fully automatic approach by means of a linear SVM-based classifier ensemble able to distinguish between up to 10 different types of pavement that appear in the Spanish roads is proposed. The optimal feature vector includes different texture-based features. The parameters are then tuned depending on the output provided by the classifier. Regarding non-crack features detection, results show that the introduction of such module reduces the impact of false positives due to non-crack features up to a factor of 2. In addition, the observed performance of the crack detection system is significantly boosted by adapting the parameters to the type of pavement.

## 1. Introduction

European countries spend large amounts of money on their road networks for reconstruction, renewal and major infrastructure repairs [1]. European road networks have kept growing in extension and the maintenance cost now accounts for a considerable part of Department of Transport budgets. In Spain 78% of last year’s road expenditure was assigned to road maintenance, of which 60% was used for road surface rehabilitation [2]. The road sector has started to consider a new approach to road maintenance by adopting a “preservation culture” [3] which consists in immediately protecting structures once they have been constructed or renewed. Hence, there are well-known economic and safety reasons and a compromise of the institutions through a preventive maintenance. However, the problem persists as there is a lack of the required precise information about the road conditions. In manual road inspection, raters go all over the road measuring its distress elements, but these surveys become too laborious and slow to allow an extensive assessment. These inspections are costly and risky for the personnel, due to traffic hazards. They also have problems associated with variability and repeatability resulting on inconsistencies in distress details. Therefore, manual road surveys do not allow one to carry out proper road maintenance. An automated distress detection system to quantify the quality of road surfaces and assist in prioritizing and planning the maintenance of the road network becomes essential. Thus, a preventive maintenance program including crack sealing operations will be feasible, achieving the goal of preserving the roads in good condition and extending their life.

Governments and institutions have made a great effort to accomplish the objective of achieving a high quality road network along the last decades. They are now, more than ever, fully aware of the need for adequate road inspection and maintenance. Nonetheless, authorities are not properly carrying out these actions because of the fact that there is still a lack of suitable methods to assess the road conditions and perform management programs both effectively and efficiently. A poorly maintained road network exposes drivers to unacceptable health and safety risks. According to the “*Sustainable Roads and Optimal Mobility*” report elaborated by the European Union Road Federation [3] the percentages of reduction in fatal accidents due to better road infrastructure only in Belgium and Sweden, are, respectively, 50% and 59%. Another report produced by the U.S. Association of State Highway and Transportation Officials [4] points out that only half of major US roads are in good condition due to costs related with additional vehicle operations (approximately 280 € per year for the average driver) as well as repair, maintenance and constructions costs (1 € spent to keep a road in good shape saves 7 € on reconstruction).

Periodic road inspections in order to have accurate and up-to-date information about the road surface condition are the most efficient way to maintain high road standards at the lowest price. Distress measurement is a crucial factor when evaluating road quality, being cracking the best indicator of the need of preventive maintenance treatments. The type, length and severity of cracks are used to quantify the road condition and identify the source of deterioration. Cracking appears in the first stages of worsening, so its detection will allow one to perform a proper maintenance, saving huge amounts of money on a later restoration. In addition, road deterioration is quite gradual in most of the road surfaces as described in [5]. Road surfaces deteriorate only 40 percent in quality during the first 75 percent of their life. Then, if not treated, the slope becomes significantly steeper as a consequence of water penetration and continued loading, and another 40 percent decrease in quality is produced in the next 12 percent of life [5]. Roads with incipient deterioration can be identified through road management programs since preventive maintenance can be applied following a cost-effective strategy (reducing the cost by a factor of 5).

## 2. Related Work

Depending on the degree of human intervention required, distress detection methods can be categorized in purely manual, semi-automated, and automated. Manual surveys have been used for long time and despite the fact that automated methods are becoming more common, they are still the most frequent methodology [6]. Human inspections present several problems, including those related to the lack of consistency among operator criteria. A great economic effort has been made by authorities and road owners to overcome the difficulties found in the developing of automated systems. Many researchers have worked on this problem, developing first semi-automatic systems to reach later fully automated ones. Semiautomatic systems use different collection technologies to grab road images and postpone the distress identification to an off-line process running in workstations, improving the safety but using still manual distress detection, or at least an important level of intervention. The identification of various distress types, as well as their severity and extent from images requires observers who have been well trained in both pavement distress evaluation and in the use of the workstations. Therefore, it is necessary to add the cost of qualified staff to the cost of expensive collection devices, discouraging agencies from adopting these technologies [7].

Different road surface distress data collection technologies are involved in the various automated commercial systems. They have been under development for the past 20 years. Initially available technologies of automated distress surveys were all based on analog video capturing and storage [8]. These systems presented low resolution and difficulty in working with computers, as the information had to be digitized to be processed, increasing the cost and complexity of the system. Along the last decade digital systems have appeared, becoming the preferred methods. Most of them employ video imaging techniques using either area scan or line scan cameras and there are just a few examples of laser-based systems. Area scan methods use a two dimensional array of pixels to cover some pavement area while line scan devices use a single line of sensor pixels to form the image by integrating successive scans. High-quality image collection required sufficient lighting to overcome shadows and sunlight.

There are quite a lot of automatic crack detection systems available with very similar characteristics, using both area scan and line scan cameras. They usually work with an image resolution that allows the detection of cracks wider than 1 mm. The differences among them are mainly the width of the scanned area, ranging from 2 to 4 m, as well as the specific distress identification algorithm applied. Here, only the most relevant systems are mentioned. In 1999 the Australian Commonwealth Scientific and Industrial Research Organization (CSIRO) was the first entity to develop an automated road crack detection system, RoadCrack [9], that was able to identify cracks wider than 1 mm at highway speed. This system has surveyed Australia for several years using line-based digital cameras mounted on a vehicle. The most widely employed system in the USA is Fugro Roadware’s Automatic Road Analyzer (ARAN) platform [10]. The ARAN system uses area scan cameras with two system configurations, 2 mm resolution with strobe lighting or 1 mm resolution with infrared lighting, to record road images that can be rated whether visually or through Roadware’s automated crack detection software (WiseCrax). Also widely employed in the USA, WayLink’s Digital Highway Data Vehicle (DHDV) [11] performs full-lane width distress surveys at speeds of up 100 km/h and uses WayLink’s Automated Distress Analyzer (ADA) software for real-time detection. In Europe, the PAVUE system [12] has operated in the Netherlands and Finland. Ramboll’s system can be equipped with either multiple video cameras or line scan cameras for collection of continuous images of the road surface which are automatically analyzed with the Automated Image Evaluation System (AIES). Finally, it is worth to mention the Highways Agency Road Research Information System (HARRIS) which is the result of 10 years research carried out by the UK’s Transport Research Laboratory [13] with the objective of demonstrating the application of state-of-the-art technology to the assessment of road conditions at traffic speed. This system has surveyed national and local roads using line scan cameras and 3.5 m survey width, providing several objective evaluations about its performance that will be discussed later on.

As an example of laser-based system we note the GIE LaserVISION system [14]. This system uses four laser sensors and provides 3D measurements giving the system the potential for improved distress measurement. Nonetheless, it has too low resolution, 3 mm by 110 mm, so it is limited to measuring transverse cracking. The most promising innovative inspection methods are evaluated in [15]. A newly developed survey system, Mobile 3D Video Mapping (M3DVM), based on a set of 360° laser scanners positioned on a van is presented. This technique needs still further development as the operation costs are too high and the location error of points in the imagery is about 5 cm. Further development is also recommended for another approach, Car as Sensor, which uses a fleet of cars equipped of sensors to collect data about road conditions. Other two systems, unmanned aerial vehicles and spaceborne techniques, are considered inappropriate for road distress detection since they present low resolution images and low flexibility sensors.

Comparing the different commercial systems is not always feasible. The understandable competitive spirit of the different manufacturers makes the information shared about their system weaknesses and the true performance minimal. Detection performance has a high dependency on the set of roads assessed. The road condition itself, the presence of non-crack elements and the different texture backgrounds faced in each case will be decisive. However, there is no public dataset with sequences of road images available so it is not possible to carry out a proper comparison. In addition, systems present different survey width making it harder to compare. In [16] it is noticed that the lack of standardized methods for evaluating the precision and repeatability of the systems constitutes a problem. Despite the harmonization efforts undertaken, different protocols for cracking evaluation are still used. Finally, the variety of levels of human intervention actually needed by the automatic systems also makes the comparison more complex. In general, commercial systems present problems identifying and quantifying cracking according to a protocol, especially those thinner than 3 mm, reaching an acceptable performance only when considering network-level tests. An investigation undertaken by the TRL [17,18] to assess the performance of five commercially available crack identification systems, including Fugro, Waylink and TRL systems, concluded that all the evaluated systems had problems with common non-crack features present on the road surface, including joints, patches, road marking(s) and road edges, resulting in much more cracking being reported than was in the reference data. Moreover, the accuracy was inconsistent, varying the performance from location to location as a consequence of the different types of road surfaces surveyed. All the systems would have difficulties in reaching the TRL requirements for acceptance, only approaching these requirements when manual intervention is used as part of the identification process. In 2008 the American Transport Research Bureau presented the 2nd Strategic Highway Research Program results [19], which shows that manual interpretation systems are widely used and automated systems are still under development in various Departments of Transport and in private companies. They consider that manual intervention should be eliminated in order to reduce cost and pointed out some limitations of current automated systems. Fatigue cracking and sealed cracks are difficult to quantify and lighting conditions and shadows cause problems. According to this report the barriers to progress are the need for better lighting systems and a better interpretation software.

This paper presents a road distress detection system that consists of an on-line images recording process and an off-line images processing stage. A vehicle equipped with line scan cameras, laser illumination and acquisition HW-SW is used to storage the digital images that will be further processed to identify road cracks. The processing module includes the phases needed to properly deal with fully automatic road distress assessment. The texture is smoothed and the linear features are enhanced by means of a pre-processing stage. Non-crack features such as joints, sealed cracks and white painting, that are usually mistakenly reported as crack features, are specifically detected and masked. A seed-based approach is then used to detect road cracks, combining Multiple Directional Non-Minimum Suppression (MDNMS) with a symmetry check. The whole detection process involves the use of several parameters that have to be adapted depending on the type of pavement to get optimal results without manual intervention. A linear SVM-based classifier ensemble trained to distinguish between up to 10 different types of pavement that appear in the Spanish roads is proposed. The optimal feature vector includes a combination of different texture-based features. The system parameters are then tuned depending on the output provided by the classifier.

The remainder of this paper is organized as follows. In Section 3 the overview of the whole road crack detection system is described in brief. Section 4 provides the description of the different system stages. Experimental results are presented in Section 5, and Section 6 concludes this paper and refers to future extensions of this research.

## 3. Overview

**Figure 1.**
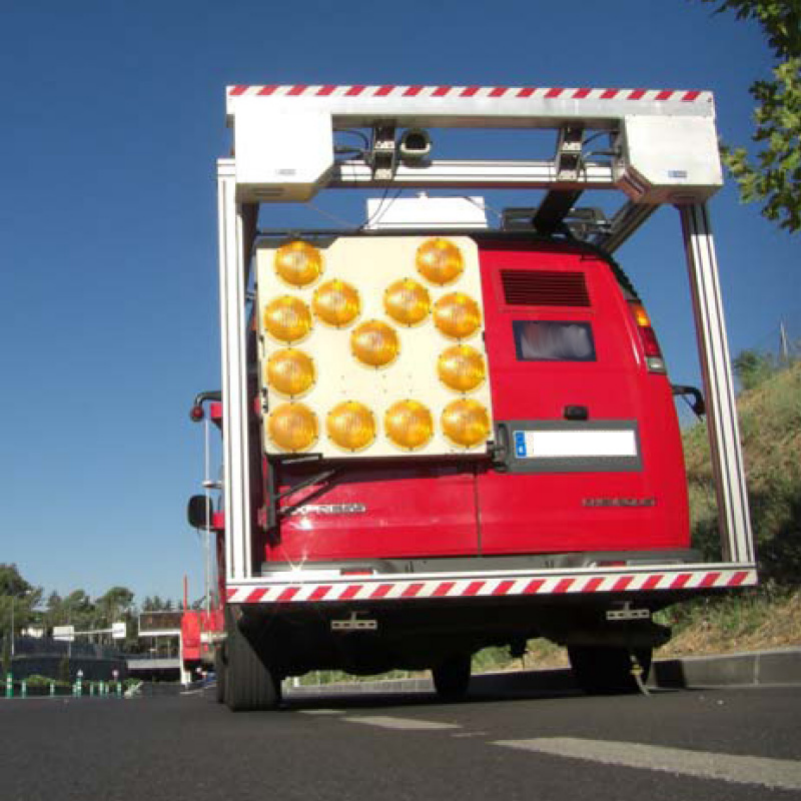
Rear of the vehicle used to acquire and store the road images.

A conveniently equipped vehicle is used to storage the images that will be further analyzed to identify road cracks. The acquisition system consists of two line scan cameras with 2,000 × 1 pixels resolution, covering up to 4 m × 1 mm of the road. A laser-based illumination module provides homogeneously illuminated images. The cameras frame rate is 28 kHz which allows the vehicle to drive up to 90 km/h. In Figure 1, a rear view of the vehicle can be seen.

**Figure 2.**
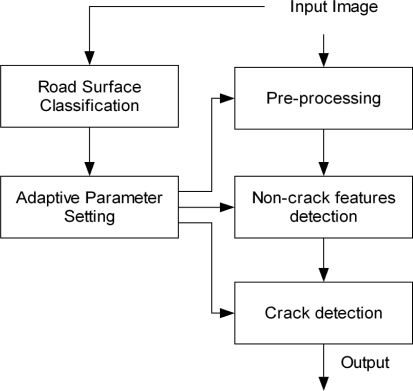
Global overview of the proposed adaptive road distress detection system that is divided into four main stages: *road surface classification*, *pre-processing*, *non-crack features detection*, and *crack detection*. The output of the classifier is used to adapt the system parameters.

A global overview of the automatic distress detection system is shown in Figure 2, where the main components are described using a block diagram. The input of the system is 4,000 × 1,000 pixels images with 1 mm of resolution in both axis. These images are obtained by integrating 1,000 images of both line scan cameras.

The pre-processing step is devised to reduce the noise as to sharpen or enhance the linear features of the raw images that usually correspond to crack features. A basic approach usually consists in a pre-processing step together with the strictly speaking distress or road cracks detection module. Nevertheless, these kind of systems incorrectly report cracking on the boundaries corresponding to non-crack elements such as joints, patches and road markings. In order to avoid these false crack detections a specific non-crack features stage is needed, masking the regions of the images where non-crack features have been detected.

The proposed adaptive road distress detection system also includes both, the non-crack features detection and the road surface classification steps. The non-crack features detection module identifies the aforementioned non-crack elements, reducing drastically the amount of false positives reported. In order to adapt the parameters of the road distress detection algorithm to the different types of pavement and road surfaces, a key stage has been here included: road surface classification.

## 4. System Description

### 4.1. Pre-Processing

**Figure 3.**
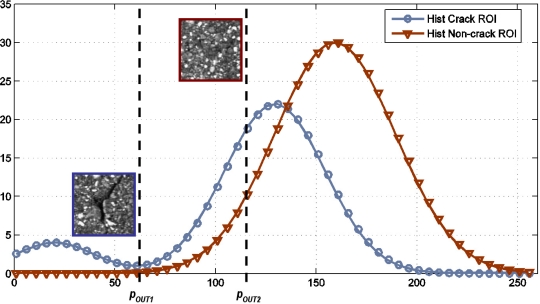
Histograms corresponding to crack area (blue) and non-crack area (red).

Pre-processing is one of the key steps of the whole distress detection system. The main idea is to ease the cracking detection process by both reducing noise and enhancing the dark linear features (possible cracks). Common pre-processing techniques are based on gray-scale morphological filters [20], image equalization [21], combinations of morphological tools and segmentation methods [22,23], and median filters [24]. Our approach is based on the assumption that the intensity of crack pixels is darker than the intensity of the pixels around the cracks. Thus, if we select a small region of interest (ROI) around a crack area, a peak will appear at the beginning of the histogram, as can be observed in Figure 2. The procedure can be summarized as follows. Using a sliding window technique, square ROIs with a pre-defined *size* (*size_pre_*) are shifted over the image. A specific *step* (*step_pre_*) is used for shifting the window. The parameter *step_pre_* will determine the size of the pre-processing image since each ROI will be represented by only one pixel. In addition, regions may overlap depending on the step parameter. For each ROI the intensity histogram *m_i_* is obtained and the cumulative histogram $M_{i}=\sum_{j=0}^{i}m_{j}$ is computed from bin i = 0 to i = 255. Once the cumulative number of observations *M_i_* reaches a pre-defined *threshold* (*th_pre_*) we store the current bin value (*p_out_* = *i)*. The threshold represents the percentage of pixels with a gray level value lower than *p_out_*. The local ROI is then replaced by one pixel (downsampling) with a gray level value equal to the current bin of the histogram (*p_out_*). Thus, local regions with crack pixels will be assigned with lower gray level values than regions without crack features. For example, as can be observed in Figure 3, the same *th_pre_* parameter will provide two different outputs: *p_out1_* and *p_out2_* that correspond to crack and non-crack sub-regions respectively, being *p_out1_* < *p_out2_*.

**Figure 4.**
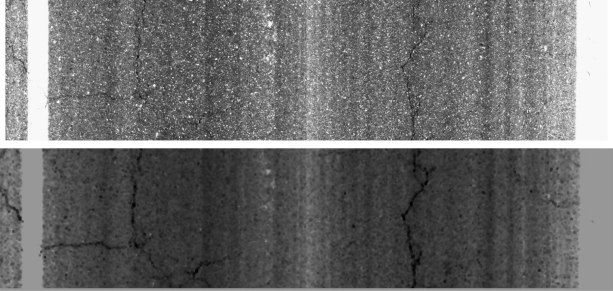
Upper row: original image. Lower row: pre-processed image.

We remark that the pre-processing step is based on three main parameters: *size_pre_*, *step_pre_* and *th_pre_*. These parameters will be adapted depending on the output (type of pavement) provided by the classification stage. In addition, it is important to note that the pre-processed image has a lower resolution than the original one, depending on the *step* value. On the one hand, further processing will be faster since the input image has a lower size and the noise is clearly smoothed. On the other hand, some details are lost due to the downsampling operation. An example of the pre-processed image is depicted in Figure 4. As can be seen, crack features are enhanced and the appearance of the non-crack areas is smoothed.

### 4.2. Non-Crack Feature Detection

Most of the distress detection systems present a high sensitivity to all features (crack and non-crack features) resulting in very few false negatives. However, too much reported cracking is usually obtained, particularly when these systems have to deal with joints, patches, white painting, sealed cracks, *etc*., providing a high number of false positive crack reports [17]. The ability to record non-crack features on the road surface is a key aspect for measuring the road surface condition, and a way of identifying false positives during crack detection. In our case, we build a specific map including the following non-crack features: *sealed cracks*, *joints* and *white painting*. This map can be used to reduce the region of interest where cracks are detected, or to mask the cracking reports.

**Figure 5.**
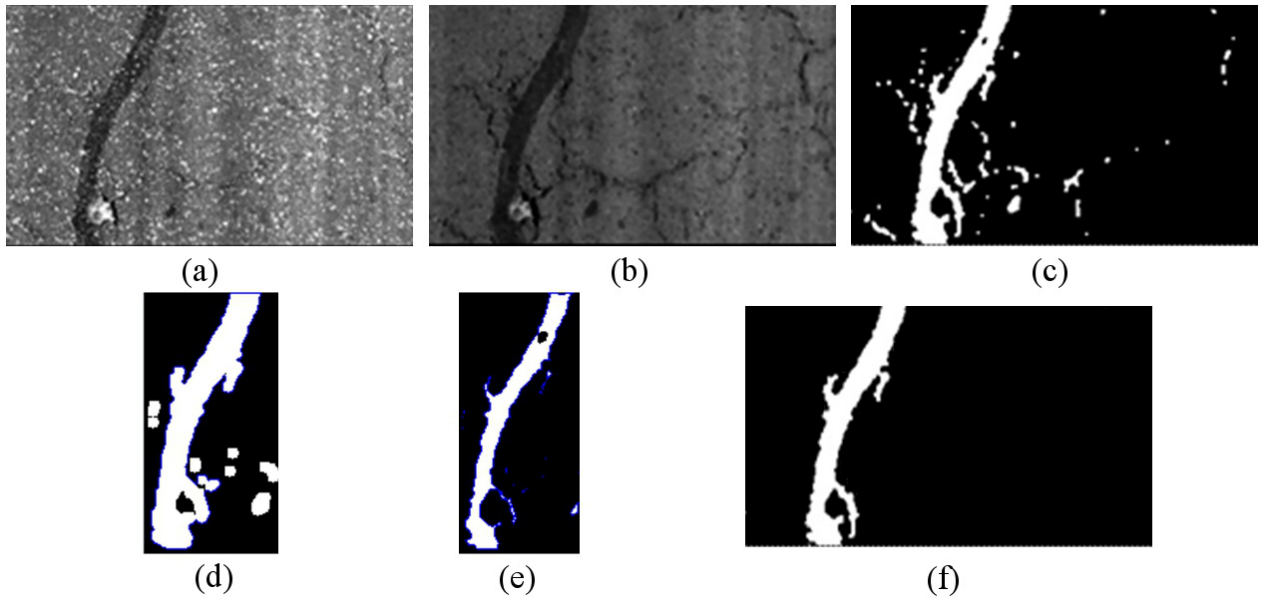
(**a**) Original image; (**b**) Pre-processed image; (**c**) Thresholded image; (**d**) Outside contour after dilate operator; (**e**) Inner contour after erode operation; and (**f**) Final result.

A very common way of repairing non critical areas of the road is to seal the cracks. Sealed cracks usually appear as wide dark lines (patches) on the images and most of the systems report false positives on the edges of the patches. In order to detect the sealed cracks we use the pre-processed image, so that computational costs are drastically reduced without losing accuracy. A basic adaptive grey-level threshold step is firstly applied. Contours are then detected over both the dilated (outside contour) and the eroded (inner contour) image. First, short contours are filtered and not taken into account. Then, the average gray-level values of the pixels corresponding to both contours (inner and outside) is computed. Our detection function is based on the fact that the pixels corresponding to the inner contour of the sealed cracks would have lower grey-level intensities than the pixels corresponding to the outside contour. Note that this assumption does not apply to crack features since cracks are much more thinner than sealed cracks, so we cannot strictly talk about inner and outer contours for cracks. More specifically, after the erode operation the inner contour of cracks does not exit. An example of the sealed cracks detection algorithm is depicted in Figure 5.

**Figure 6.**
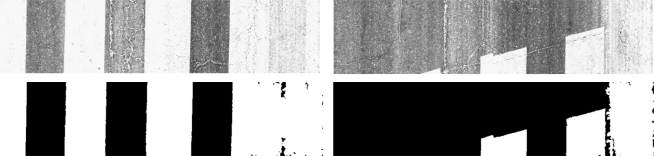
White painting detection examples. Upper row: downsized original images; Lower row: white paining detection results. Note that paint cracks are not surface cracks.

White painting is another common source of false detections that appears in road markings such as white lines, white arrows, text on the road, *etc*. Linear features appear due to paint cracks, that do not correspond to real cracking. The white painting detection process does not use the pre-processed image since white pixels are smoothed. Instead, a downsized version of the original image is used to reduce computational costs, and again, without losing accuracy. A basic gray level threshold (non-adaptive) is applied and contours are detected and filtered. The filtering process uses the following features: contour area, bounding box area, fitted bounding box area, contour orientation and the aspect ratio. The idea is to detect blobs with high rectangularity and wide area inside the bounding box. Some examples can be seen in Figure 6.

Concrete surfaces present longitudinal joints which are construction joints between adjacent lanes that have been paved separately, and transverse joints which can be either construction joins separating layers paved at different times or contraction joints placed at recurrent intervals. Longitudinal joints appear as vertical straight lines located close to one of lane edges preventing longitudinal cracking from spreading. Transverse joints appear as horizontal straight lines crossing the lane and controlling transverse cracking. Joins are dark linear features and they have a similar gray-level histogram distribution and shape than cracking features. Therefore, they are likely to be incorrectly considered as cracking by crack detectors and they must be detected and their surrounding pixels masked. In addition, joints are characteristics of concrete surfaces, so identifying them will help us in the road classification task by using a hierarchical approach. The joint detector uses a downsized image as input. High intensity pixels are saturated and the edges of dark elements are obtained by means of the Canny edge detector. Non-straight line segments are filtered out by applying horizontal and vertical restrictions on the contour’s shapes. This filtering step avoids the detection of false joints and reduces the computational load required for further steps. Two images are obtained, the first with horizontal segments, and the second with vertical ones. Then, straight lines are extracted by means of the Hough transform. A straight line in the image plane can be defined in polar coordinates with a distance to the origin (left-up corner) *ρ*, and the angle between the normal line and the abscissa axis *θ*:

(1) $$x_{i}\cdot \text{cos}(\theta )+y_{i}\cdot \text{sin}(\theta )=\rho$$

**Figure 7.**
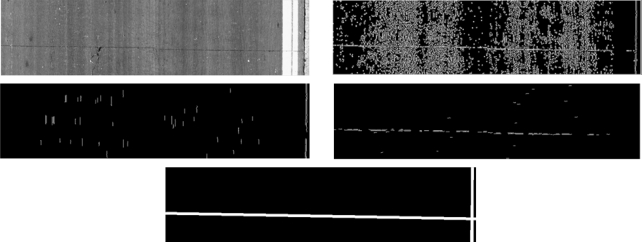
From upper row to lower row: downsized original image, Canny image, image with vertical segments, image with horizontal segments, Hough transform result.

The parameter space, *p = (ρ, θ)*, must be quantized and expressed in a 2D accumulation matrix *a*, whose elements are initially set to zero. So, an element *a(ρ, θ)* is increased by 1 for every contour point (*x_i_y_i_*) in the image-domain. Different algorithms have been proposed in order to decrease the computational time of the Hough transform. A multi-dimensional quadtree structure for accumulating is suggested in [25] (coarse-to-fine method). In [26] a method based on the fact that single parameter space point can be determined uniquely with a pair, triple or generally n-tuple of points from the original image (many-to-one mapping method) is proposed. In our case, a constrained accumulation matrix *a* is used as a method to decrease the computation time. The aim is to search for lines using two specific constraints. On the one hand, horizontal lines are detected in the image filtered with horizontal segments with the following restriction in the accumulation matrix: *θ* *= 90° ± 5°*. On the other hand, vertical lines are detected in the image filtered with vertical segments with the following restriction in the accumulation matrix: *θ* *= 0° ± 5°*. Accumulator threshold is fixed proportional to the width and the height of the image respectively. In addition, the region of the image where vertical joints can be detected is restricted to one third of the image. Finally, a binary image with both types of joints is obtained (see Figure 7).

### 4.3. Road Surface Classification

A road surface classification step is needed due to the high diversity of road surfaces that appear in real environments. It is extremely difficult to develop a monolithic road distress detection system able to provide reasonable results using the same parameter settings independently of the type of pavement. Accordingly, we have developed a road surface classification stage to classify road surfaces into different classes allowing to triggering of the road distress detection algorithm with a pre-defined set of parameters that are optimal for the specific type of pavement.

**Figure 8.**
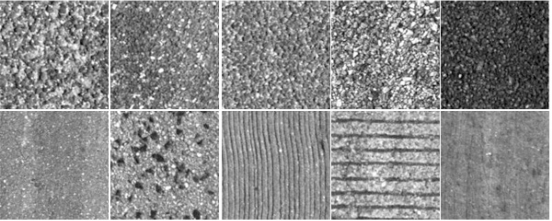
Surface classes: seven classes are bituminous (asphalt) materials and three are concrete.

The number of classes is chosen by visual inspection based on the type of materials used to build the roads as well as the degree of granulation and striation. An example of each one of the 10 classes considered is depicted in Figure 8. Seven classes are bituminous (asphalt) materials and three of them are concrete.

The surface classification process is carried out using a multi-class SVM [27] based on the standard one-*versus*-one approach to reduce the single multi-class problem into multiple binary classification problems. The maximum-margin hyper-plane which best separates the 10 classes is computed by solving an optimization problem. A linear kernel function has been chosen as it achieves the best accuracy when comparing with polynomial, radial basis and sigmoid kernel functions. In addition, the linear kernel function provides an on-line application function that involves a dot-product with the weight vector independent of the support vectors.

A set of up to 20 square sub-regions of 256 × 256 pixels size are used to perform the classification by majority voting decision. Sub-regions must be placed in the central columns of the image and avoiding the use of pixels masked as non-crack features.

Different feature extraction methods, settings and feature group combinations have been evaluated in order to choose the most suitable trade-off between classification performance and computational load. It is important to maintain a low computational cost so that the application usage remains feasible in real exploitation scenarios. Some feature extraction algorithms, such as Gabor Filters [28] and Wavelets [29], have been tested and discarded, as their inclusion does not provide a significant performance improvement. The main components of the feature vector are obtained using Gray-Level Co-occurrence Matrix (GLCM) [30], Maximally Stable Extremal Regions (MSER) [31] and Local Binary Patterns (LBP) [32]. In order to take advantage of the different gray-level histogram distributions of each type of surface, four statistics are also computed from the gray-level histogram: average, deviation, skewness and kurtosis. Finally, two components are obtained using the 1-D Fast Fourier Transform (FFT) over four equidistant rows and over four equidistant columns.

The GLCM, introduced by Haralick [30], stores how often the different combinations of pixel gray-levels occur in the image, separated by a particular distance in a specific direction. The most common setting considers 1 pixel distance and four angles or pixel relationship directions (0°, 45°, 90° and 135°). In our approach, four angles are also considered but up to four pixel distances (1, 3, 5 and 9) are evaluated (GLCMM), resulting in 16 different matrices. An alternative using a pyramid image reduction approach has been also evaluated (GLCM-pyr). Haralick defined a set of 14 texture measurements based on this matrix, from which only four have been considered relevant to the surface classification problem: homogeneity, entropy, contrast and energy. The GLCM calculation requires an image quantification step in order to reduce both the computational load and the vector dimension. Different quantification levels have been tested concluding that no significant improvement is reached with more than 20 quantification levels.

**Figure 9.**
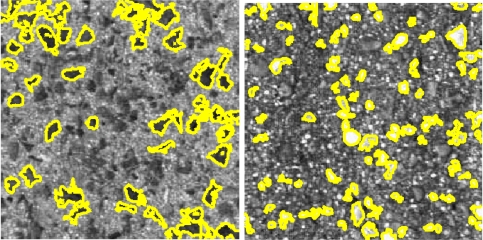
MSER detection results. **Left**: black blobs. **Right**: white blobs.

The different types of pavements present different grades of granulation, varying in size and gray-level, as well as in their distribution. The granulation results on either rougher or smoother surfaces. Therefore, blobs detection may help to improve the classification performance. A solution based on MSER detection has been implemented. Although the MSER algorithm was originally proposed in [31] as an approach to solve the wide-baseline stereo problem, it has also been demonstrated to be a powerful blob detector, since it presents invariance to monotonic transformation of image intensities and allows multi-scale detection. An extremal region is a set of connected components that maintains the intensity levels of them below a threshold. Maximally extremal regions are extremal regions which satisfy a stability criterion. Minimal extremal regions are then detected by inverting the original image, so dark and bright blobs are both detected. Regions are filtered according to their shape, so only regions that satisfy circular shape restrictions are considered. Two blob size ranges are defined. Three statistics are computed for each one of the ranges and gray-levels: the number of blobs, the averaged size and total area. Figure 9 shows white and black blobs detection result examples.

Local Binary Pattern (LPB) is a gray-scale and rotation invariant texture operator introduced by Ojala [32]. LBP is built by thresholding pixels in a 3 × 3 window including its central pixel. If the pixel value is greater or equal than the central pixel, it is set to 1; otherwise it is set to 0. LBP codifies the central pixel as the sum of the pixel values weighted according to their position around the central pixel. Some extensions have been tested. For example, the variation of the distance of the samples (pixels around the central one) or the number of samples have not shown significant improvement in the performance. Histograms formed only by uniform patterns (that contain at most two bit-wise transitions both from 0 to 1 and vice versa), are usually computed (LBP-u). A common extension is to consider rotational invariant patterns together with the uniform ones (LBP-riu). Besides, LBP is also performed on the gradient image in both directions, horizontal and vertical (LBPG).

### 4.4. Road Distress Detection

Cracks appear in road images as linear features darker than their surrounding pixels. They are nearly continuous with a dominant orientation. Cracks can be thinner than aggregates but they are always longer than them [22]. Taking these general cracks characteristics into consideration, a large amount of different crack detection methods have been previously implemented. One obvious first approach is to apply a gray-level threshold to obtain a binary image with crack and non-crack pixels. Most of these approaches are based on the Otsu’s thresholding method from gray-level histograms [33]. Different implementations and approaches based on [33] have been proposed [34–38]. These methods are usually improved using morphological operations [21,22,39–41]. Thresholding methods are very simple but they usually provide noisy results. Other approaches consider that crack information is mostly contained in the high frequencies components (transform-based). Most common transform-based approaches for crack detection applications are Wavelet Transform [20,24,42–45], Contourlet Transform [46,47] and Beamlet Transform [48,49]. More sophisticated approaches propose the use of classifier ensembles to deal with the road crack detection problem. The image is divided into several cells focusing on distinguishing between crack and non-crack cells. Most used approaches are Neural-Network-based [50–54], Support Vector Machine-based [55], and k-nearest-neighbors-based [56]. The main drawback of these methods is the supervised learning nature which requires a considerable amount of samples to have a good representation of the real environments.

The method proposed in this paper can be classified in a different group of methods known as seed-based approaches [57–62]. These methods share two common steps. First, points likely to belong to a crack feature are extracted (seed points). Second, paths between seeds are obtained joining seeds by means of path growing techniques.

**Figure 10.**
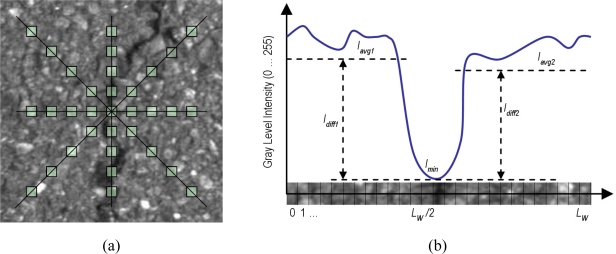
(**a**) The four linear windows at 0°, 45°, 90° and 135°; and (**b**) Symmetry profile of a cross section of a linear feature including the parameters involved in the symmetry check.

A Multiple Directional Non-Minimum Suppression (MDNMS) combined with a symmetry check described in [57] for linear feature detection is used to obtain the seeds. Non-minimum suppression is a process for marking all pixels whose intensity is not minimal as zero within a certain local neighborhood. This local neighborhood is here defined as multiple directional linear windows at angles of 0°, 45°, 90° and 135° (see Figure 10(a)). As suggested in [57] additional directions can be used. However only four directions are used since we have not reported a significant improvement of using eight directions instead of four. The length of the linear window (*L_W_*) is an important parameter that has to be adapted to the size of the images to avoid multiple local minimums. As in [57] we use the union of the outputs of multiple directional non-minimum suppression for seeds detection. In addition, the cross feature gray-level intensity profile of the linear feature at a particular position is roughly considered symmetric around the minimum point (with intensity *I_min_*). As depicted in Figure 10(b), several parameters are used to check whether a local minimum is along a linear feature in the image for a particular direction (linear window). These parameters are described by Equations (2) and (3):

(2) $$I_{\mathrm{avg}1}=\frac{1}{L_{W}}\sum_{i=0}^{L_{W}/2-1}I(i);\;I_{\mathrm{avg}2}=\frac{1}{L_{W}}\sum_{i=L_{W}/2+1}^{L_{W}-1}I(i)$$

(3) $$I_{\mathrm{diff}1}=I_{\mathrm{avg}1}-I_{\text{min}}\;;\;I_{\mathrm{diff}2}=I_{\mathrm{avg}2}-I_{\text{min}}$$

*I_min_* is the gray-level intensity of the local directional minimum. *I_avg1_* and *I_avg2_* are the average gray-level values of half linear windows on the two sides of the local minimum. *I_diff1_* and *I_diff2_* are the differences between the two average gray-level intensity values and the minimum value. For a linear feature in the image both of the *I_diff1_* and *I_diff2_* values should satisfy the following condition:

(4) $$I_{\mathrm{diff}1}\geq {\mathrm{th}}_{\mathrm{SymDiff}}\;;\;\;I_{\mathrm{diff}2}\geq {\mathrm{th}}_{\mathrm{SymDiff}}$$

where *th_SymDiff_* is a threshold parameter adaptive to the type of pavement. This value, as well as the length of the linear windows, will be adapted according to the output provided by the classifier. Note that if only one of the difference values is larger than the threshold, the location of the local minimum resembles more like a step edge rather than a linear feature.

**Figure 11.**
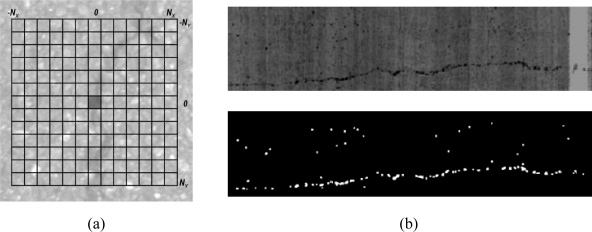
(**a**) Sliding window parameters; and (**b**) **Upper**: pre-processed image. **Lower**: mask with possible seed locations.

In order to avoid triggering the seed detection process at all pixels locations, seeds detection is only applied in pre-computed regions of interest (ROI). A local sliding window (2*N_x_* + 1, 2*N_y_* + 1) is used at all pixel locations of the pre-processed image. The average gray-level value (*I_avg_*) of the local window is compared with the gray-level value of the central pixel (*I_c_*) (see Figure 11(a)). The following condition has to be met:

(5) $$I_{C}=I(0,0);\;I_{\mathrm{avg}}=\frac{1}{\left(2N_{X}+1)(2N_{Y}+1\right)}\sum_{i=-N_{X}}^{N_{X}}\sum_{j=-N_{Y}}^{N_{Y}}I(i,j)$$

(6) $$I_{C}\leq K_{S}\cdot I_{\mathrm{avg}}$$

If the condition described in Equation (6) is met, the pixel will be masked. Otherwise, the pixel will be discarded (as possible seed). Note that parameter *K_S_* will be adapted according to the type of pavement. An example of the masked pixels provided by this step can be seen in Figure 11(b).

**Figure 12.**
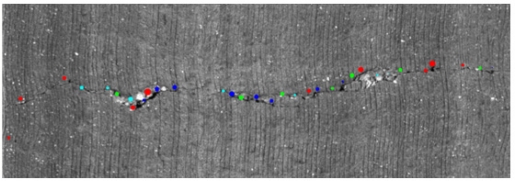
Seeds detection results. The color indicates the orientation. The thickness of the circle used to draw the seeds is directly proportional to the seeds thickness.

Once the MDNMS combined with the symmetry check is applied only in the masked pixels, we finally obtain the seeds. This process allows us to maintain relevant information for each one of the seeds: seeds orientation and seeds thickness. The orientation is directly obtained from MDNMS method. The thickness is roughly estimated using the cross feature gray-level intensity profile of the linear feature (Figure 10(b)). Figure 12 depicts an example of the output provided by the seeds detection method.

**Figure 13.**
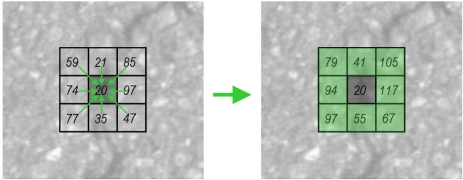
Initialization step. **Left**: backtracking information. **Right**: distance costs of the 8-connected pixels around the seed.

**Figure 14.**
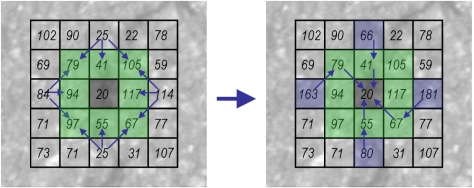
Iterative process: distance cost computation of pixels located on *x-y*-axis. **Left**: possible neighbor pixels locations. **Right**: distance costs and backtracking information.

**Figure 15.**
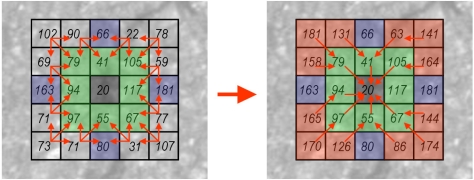
Iterative process: distance cost computation of pixels located on quadrants. **Left**: possible neighbor pixels locations. **Right**: distance costs and backtracking information.

The second stage of seed-based methods is the linking process to connect the seeds (path growing). Several approaches have been previously proposed in the literature. In [59,60] seed points are connected by proximity. The authors of [62] proposed a percolation method based on the natural phenomenon of liquid permeation. In [58] a normalized distance cost polar map of circular regions is used and paths are obtained based on a backtracking process. This approach implies two transformations (from Cartesian to polar and vice-versa) that have a high computational cost, and only allows paths to grow proportionally to the radius of the circles used, which is not always adequate. Our path growing method is based on the backtracking idea proposed in [58] but adapted to directly work in Cartesian coordinates using square regions instead of circular ones. A minimum distance cost map is computed for all the seeds collecting the lowest cost from the center of the region to the rest of the pixels, as well as the backtracking information (previous pixel location in the path with the lowest cost). Based on the fact that crack features are dark linear features, the gray-level value is directly used when computing the distance cost map. The process starts initializing the 8-connected pixels of the seed (see Figure 13). Then, an iterative process is triggered. We firstly obtain the minimum distance cost of the pixels located on the *x*-*y*-axis. As can be observed on the left side of Figure 14, only a pre-defined number of neighbors are evaluated. These neighbor locations are previously defined depending on the quadrant. Finally, the minimum distance cost of the pixels located on the different quadrants of the square region is calculated (see Figure 15). Note that the costs of the corner pixels are calculated at the end of the iteration. The pseudo code for obtaining this minimum distance cost map is given in Algorithm 1. The number of iterations of the process is directly related with the width of the square region around the seed (variable *L* in the pseudo code presented in Algorithm 1).

**Algorithm 1.**
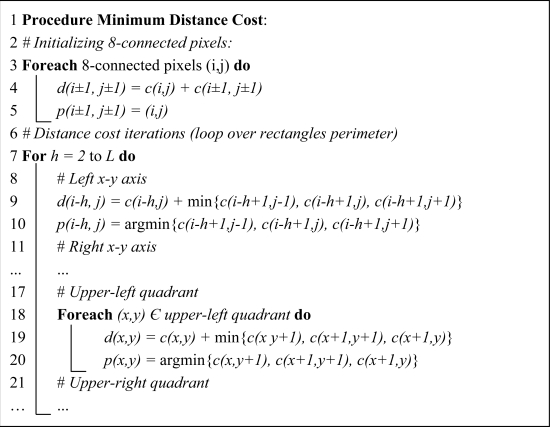
Pseudo code of the distance cost procedure.

In the above code, *d(i,j)* is the minimum distance cost for the *(i,j)* pixel and *p(i,j)* is the previous pixel location in the minimum distance cost route from pixel *(i,j)* to the center of the square region. This process involves the collection of the current best cost at a pixel and its backtracking information. Once the minimum distance cost map is obtained, we find other seeds in the map and perform the backtracking to find the paths between seeds. Backtracking is carried out by using the previous pixel locations stored in *p(i,j)* from the found seeds to the central pixel of the region.

The proposed *ad hoc* solution outperforms the ordinary dynamic programming (DP) technique for solving the shortest path problem in terms of computational costs and it needs less storage requirements. Although DP is a well-established technique, the method is somewhat limited from a computational point of view due to its recursive nature. Both storage and computational requirements are critical factors in the context of road crack detection, specially in production schemes.

**Figure 16.**
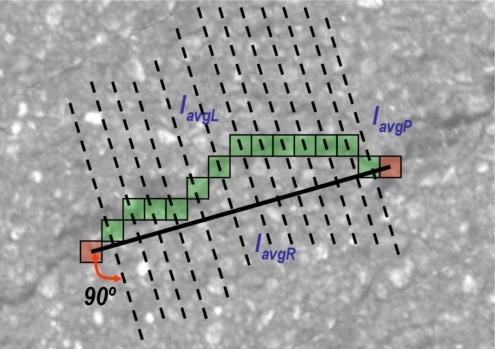
Path quality check. Linear windows are orthogonal to the orientation between the seeds (seeds are depicted in red and pixels of the path are depicted in green).

The obtained paths are checked to make sure that the average intensities along, left of the path and right of the path, meet certain symmetry conditions [58]. Non-minimum suppression along the path is assured thanks to the minimum distance cost computation. However, cracks are characterized by an approximately symmetric intensity profile across the linear feature (opposed to edges that show a step profile). This translates the problem into approximately equal average gray-level intensity values on either sides of the path. We apply the same symmetry restrictions described by Equations (2–4) at all the pixels of the path between two seeds. The linear window used at each pixel is orthogonal to the orientation between both seeds (see Figure 16). *I_avgP_* is the average intensity of the pixels along the path. *I_avgL_* and *I_avgR_* are the average intensity values along the left and right of the path. For a linear feature the following conditions have to be met:

(7) $$I_{\mathrm{avgP}}\leq {\mathrm{th}}_{\mathrm{PATH}}$$

(8) $$I_{\mathrm{avgL}}-I_{\mathrm{avgP}}\geq {\mathrm{th}}_{\mathrm{SymDiffPATH}}\;;\;\;I_{\mathrm{avgR}}-I_{\mathrm{avgP}}\geq {\mathrm{th}}_{\mathrm{SymDiffPATH}}$$

Both, *th_PATH_* and *th_SymDiffPATH_* are threshold parameters adaptive to the type of pavement. Paths that pass the quality check need to be masked out in order to avoid using them when finding paths between other seeds. This process is repeated until no more seeds are available.

### 4.5. Adaptive Road Distress Detection

The proposed road distress detection method described above contains a total of 8 parameters that have to be fine-tuned in order to have an appropriate behavior. These parameters are described by the following vector *Q = {size_pre_*, *step_pre_*, *th_pre_*, *L_W_*, *th_SymDiff_*, *K_S_*, *th_PATH_*, *th_SymDiffPATH_}*. The first three parameters correspond to the pre-processing stage. The rest of the parameters are related to the crack detection algorithm. A specific set of parameters can work perfectly with a specific type of pavement but provide very noisy results with other type of pavement surfaces. Accordingly, these parameters have to be adapted with respect to the output provided by the classifier stage. The issue of automatic system parameter optimization has so far not been covered in the context of road crack detection, to our knowledge. A large amount of literature exists on numerical methods dealing with minimization of non-linear objective functions. Such approaches are impractical for the given problem because the number of parameters involved is relatively high and a single evaluation of the objective function is computationally expensive. Instead we have carried out a manual, supervised procedure for parameter setting by evaluating the results of the system with a set of pre-selected images corresponding to the 10 classes of pavement. More specifically, a two-level grid-search approach is proposed in this work. First, the most sensitive parameters are optimized to assure that an adequate number of seeds are selected. These parameters are *size_pre_*, *th_pre_*, *K_S_*, and *th_SymDiffPATH_*. Results are ordered by recall and only the sets located in the first positions are evaluated in the second step, where the remaining parameters are obtained. In this way, the output of the SVM-based classifier is directly used to select the parameter vector *Q_i_*, *i = 1*, ..., *10* that will be finally passed to the road crack detection module. A more sophisticated and automated optimization approach is out of the scope of this paper and it is left for future work.

## 5. Experimental Results

In this section we firstly describe the classification results comparing the different group of features used in the optimization process. Then non-crack features detection results are presented, including how this stage affects to the performance of the road crack detection system. Finally, we present the adaptive road crack detection results.

### 5.1. Classification Results

**Figure 17.**
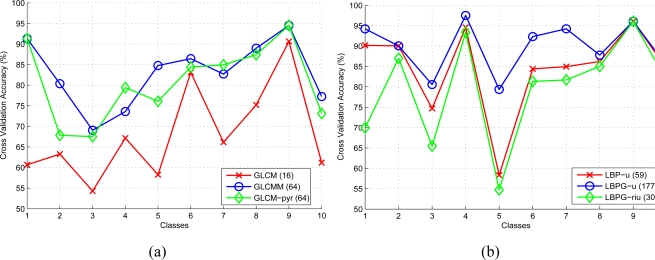
Cross validation accuracy (%) of (**a**) GLCM and (**b**) LBP configurations. Note that labels include the vector dimension.

A 10-fold cross validation algorithm over a data set of 9,000 images (900 images of each class) of 256 × 256 pixel size is used to assess the performance of the SVM-based pavement classifier for different feature extraction methods combined in different groups. In a first experiment we obtain the results of the different GLCM and LBP features working alone. Figure 17(a) depicts the cross validation accuracy among the different GLCM implementations used. Figure 17(b) shows the performance results of the different LBP configurations. Best results are obtained using GLCMM and LBPG-u features with dimensions of 64 and 177 respectively.

**Figure 18.**
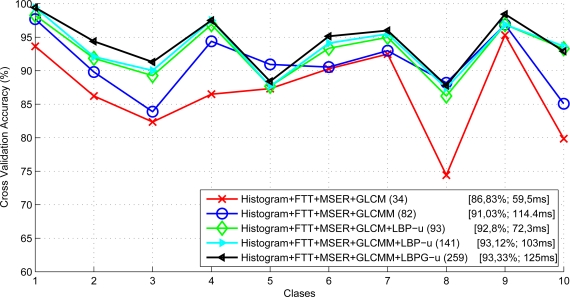
Cross validation accuracy (%) of different feature groups. The legend includes information about the vector dimension, average accuracy and computational cost.

In a second step, we evaluate the accuracy of different groups of features combining MSER, Histogram and FFT derived statistics with the features obtained from the different GLCM and LBP implementations. As can be observed in Figure 18 the optimal feature vector configuration is composed of the following features: *Histogram, MSER, FFT, GLCMM, LBPG-u*. Note that this feature vector provides the best level of accuracy of 93.33% at the highest computational cost of 125 ms (the computational cost depicted in Figure 18 corresponds to the feature computation plus the linear-SVM evaluation). The confusion matrix for the selected optimal vector configuration is shown in Table 1.

Note that these are single-frame classification results. In practice, the classifier is triggered using 20 sub-regions of 256 × 256 equally distributed along images of 4,000 × 10,000 pixel size (4 × 10 m). The so-called *majority voting scheme* is used to assign the final result as the class which has the largest number of votes.

### 5.2. Non-Crack Feature Detection Results

As described above, non-crack features are prone to report false cracking. In order to quantify the magnitude of the effect of false cracking reported due to non-crack features a specific set of 1.102 images of 4,000 × 1,000 pixel size (4 × 1 m) with considerable cracking has been selected, including features such as sealed cracks, joints and white painting. Crack and non-crack features have been manually labeled to obtain the corresponding ground truth.

In a first experiment we compute the accuracy of our non-crack features detection stage by comparing the results with the ground truth. A detection by the non-crack features detection system is counted as correct (true positive) if the coverage $\Gamma =\frac{A(a\cap b)}{A(a\cup b)}$ of a detected non-crack feature *a* and a non-crack feature label *b* is above 0.6. Table 2 summarizes the detection results for joints, white painting and sealed cracks (patches) independently.

**Figure 19.**
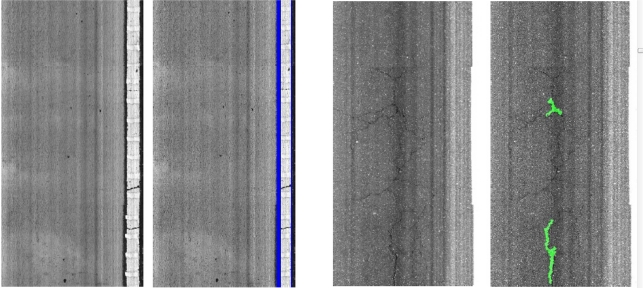
Non-crack false positives. **Left**: dark and narrow longitudinal texture band detected as joint. **Right**: wide cracks detected as sealed cracks.

False positives and negatives occur due to different road image conditions. Most of the false joints arise from dark and narrow longitudinal texture bands, which do not influence the reported cracking. However, most of the false patches result from wide cracks that will be masked and not considered by the crack detection module. Some examples of the false positives can be seen in Figure 19.

**Figure 20.**
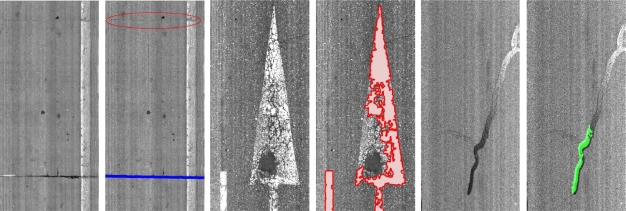
Non-crack false negatives. **Left**: not detected joint due to misalignment between images. **Center**: paint deterioration. **Right**: sealed crack not detected due to reflections.

Concerning false negatives, white painting is occasionally not detected due to paint deterioration. Most of the false negatives reported by the sealed cracks detector appear due to reflections and overexposure. Finally, joints are usually not detected because most of their pixels are not visible in the images (images alignment, line scan cameras integration. *etc*.). Figure 20 depicts some false negatives examples.

**Figure 21.**
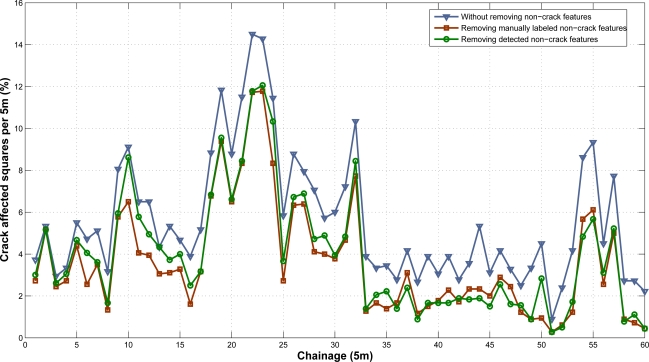
Computed percentage of each 5 m subsection of road affected by cracking, without removing non-crack features, removing manually labeled non-crack features and removing non-crack features detected by the system.

The adaptive road crack detection system is triggered and the results are masked removing cracking from both non-crack features labeled (ground truth) and detected (system results). In order to show the results in a manageable format, we use the same procedure proposed in [17] when comparing the performance of up to seven different commercial systems. A crack grid is obtained by placing 200 mm grid squares over the images. The grid squares containing cracking are filled in, and the number of crack grid squares over 50 m length are summed to obtain the areas of cracking identified by the system. In this case, we use 5 m subsections instead of 50 m subsections. Thus, the percentage of each 5 m subsections of the road is given by Equation (9):

(9) $$C_{5m}=100\cdot \left(\frac{G_{D5m}}{G_{T5m}}\right)(\%)$$

where *G_D5m_* refers to the number of 200 mm × 200 mm grid squares containing cracks and *G_T5m_* is the total number of 200 mm × 200 mm grid squares in 5 m subsection. Some of these results are depicted in Figure 21 (for visualization purposes we only show the first 300 images). The Root Mean Square Error (RMSE) between the cracking reported with and without using non-crack feature detection with respect to the cracking after masking them with the labeled non-crack features is 0.0235 (2.35%) and 0.0112 (1.12%) respectively. In other words, the non-crack feature detection module reduces the impact of false positives due to non-cracking features by a factor of 2.

### 5.3. Adaptive Road Distress Detection Results

**Figure 22.**
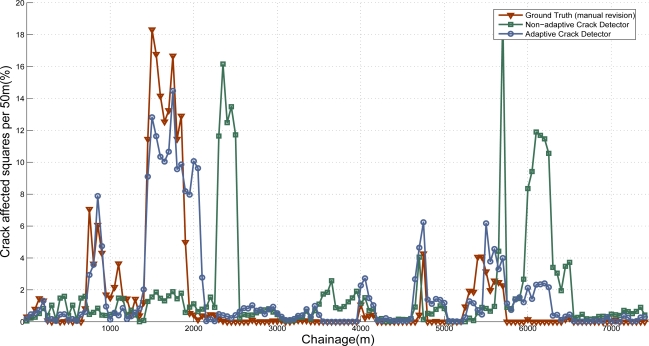
Computed percentage of each 50 m subsection of road affected by cracking. Comparison between the ground truth, crack detection system and adaptive crack detection system.

**Figure 23.**
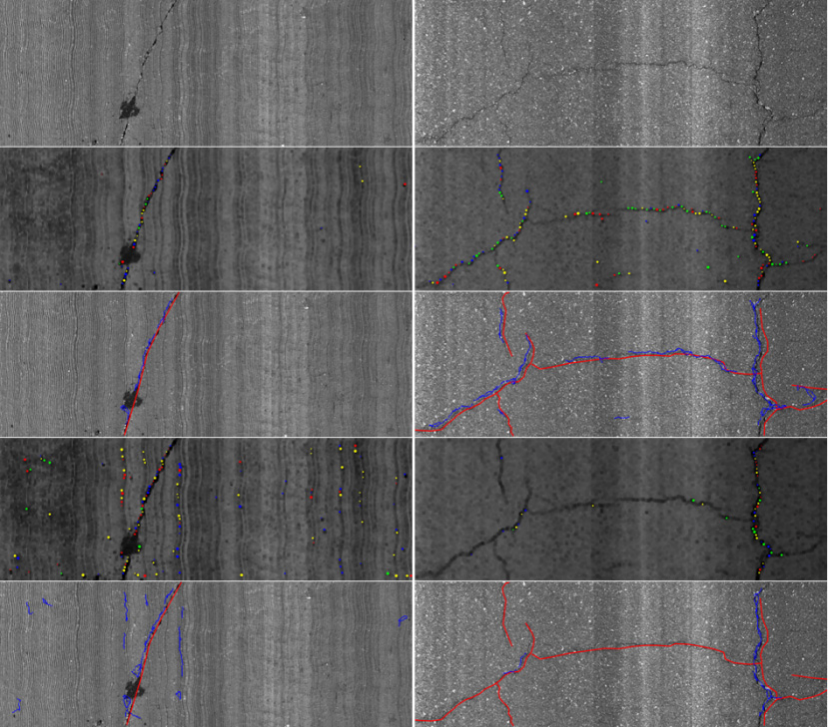
From upper to lower row: original image, seeds results with adaptive parameters, cracking results with adaptive parameters (ground truth in red; cracking in blue), seeds results with fixed parameters, cracking results with fixed parameters. **Left column**: example with false positives reported; **Right column**: example with false negatives reported.

We have collected a total of 7,250 images (4 × 1 m) from 30 different Spanish roads, containing cracked regions with the 10 different types of pavements considered in this work (725 images of each class). A manual labeling process has been carefully carried out to obtain the ground truth with the location of the cracks in the images. On the one hand, we obtain the results of the crack detection system with a fixed set of parameters tuned to provide the best results possible. More precisely, the system was triggered 10 times, each one with the parameter settings that correspond to a specific surface class. RMSE is obtained for each case and we use the parameters of the class that provides lower RMSE. On the other hand, the adaptive road crack detection system is triggered setting the parameters according to the output provided by the SVM-based classifier. Note that the images contained in the data set used to train the classifier (Section 5.1) are totally different from the images used to assess the performance of the adaptive road crack detection system. The use of independent dataset avoids overfitting. Again, in order to show the results in a manageable format, we use the same procedure proposed in [17]. In this case we use 50 m subsections, so the percentage of each 50 m subsections of the road is computed as:

(10) $$C_{50m}=100\cdot \left(\frac{G_{D50m}}{G_{T50m}}\right)(\%)$$

where *G_D50m_* refers to the number of 200 mm × 200 mm grid squares containing cracks and *G_T50m_* is the total number of 200 mm × 200 mm grid squares in 50 m subsection The results are presented in Figure 22. As can be observed, thanks to the use of the classifier to select a proper set of parameters depending on the type of pavement, there is very good agreement between the ground truth and the adaptive crack detection system assessments, including peaks and areas without cracking. On the contrary, if system parameters are not adapted, some peaks with reported cracking arise from false positive features. Some examples corresponding to both non-adaptive and adaptive crack detection systems are presented in Figure 23. It is remarkable the amount of false positives that appear due to bad parameter setting. The RMSE of the non-adaptive crack detection system is 0.0493 whereas the RMSE of the adaptive approach is 0.019 (reduced by a factor of 2.59).

## 6. Conclusions

This paper presented a road crack detection system that provides fully automatic road distress assessment. A vehicle equipped with two line scan cameras, a laser illumination system and acquisition HW-SW is used to storage the digital images that will be further processed by means of image processing techniques to identify road cracks in an off-line process. Pre-processing is firstly carried out to both enhance the linear features that may correspond with cracking and smooth the texture of the pavement to ease the detection process. An histogram analysis is performed to that effect, resulting in a low size image. Non-crack features detection is then applied to specifically separate areas of the images with joints, sealed cracks and white painting, that usually generate false positive cracking. A seed-based approach is proposed to deal with road crack detection Firstly, a Multiple Directional Non-Minimum Suppression (MDNMS) combined with a symmetry check is used to obtain the seeds. Then, a minimum distance cost map is computed in Cartesian coordinates for all the seeds collecting the lowest cost from the center of a square region of a pre-defined size to the rest of the pixels, including backtracking information (previous pixel location in the path with the lowest cost). Thus, seeds can be easily linked into paths that have to meet certain symmetry conditions. The whole detection process involves the use of several parameters, and their correct setting becomes a key component to get optimal results without manual intervention. A fully automatic approach is proposed by means of a linear SVM-based classifier ensemble able to distinguish between the 10 different types of pavement that appear in the Spanish roads. The optimal feature vector includes different texture-based features. The parameters are then tuned depending on the output provided by the classifier.

On the one hand, regarding non-crack features detection, results show that the introduction of such module reduces the impact of false positives due to non-cracking features up to a factor of 2. On the other hand, the observed performance of the crack detection system is significantly boosted by adapting the parameters to the type of pavement.

Future work concerns improvements in the classifier ensemble, such as hierarchical classification using joints to differentiate between concrete and non-concrete surfaces, boosting process with much more samples, new features, and multi-frame validation. An experimental comparison is expected to be done by implementing other crack detection approaches so as to compare the actual performance of our system. New performance indexes can be computed to differentiate from longitudinal, transversal and alligator cracking. As stated in [63] the definition of the indexes is critical to obtain useful results for the end user (road administrators). More sophisticated automatic system parameter optimization procedures will be explored. Finally a huge database with millions of images corresponding to thousand of kilometers will be used, with its corresponding ground truth, to validate the system as a feasible tool for exploitation purposes.

## Figures and Tables

**Table 1.** Confusion matrix for the optimal feature vector configuration.

**Classes**	**1**	**2**	**3**	**4**	**5**	**6**	**7**	**8**	**9**	**10**
**1**	**98.27**	0.00	1.73	0.00	0.00	0.00	0.00	0.00	0.00	0.00
**2**	0.00	**91.84**	2.04	1.53	0.00	4.59	0.00	0.00	0.00	0.00
**3**	0.77	0.26	**89.26**	0.51	7.93	1.28	0.00	0.00	0.00	0.00
**4**	0.00	1.04	1.04	**86.88**	0.83	0.21	0.00	0.00	0.00	0.00
**5**	0.00	2.17	4.35	0.72	**87.68**	5.07	0.00	0.00	0.00	0.00
**6**	0.00	3.07	1.79	0.00	1.02	**93.95**	0.26	0.26	0.00	0.26
**7**	0.75	0.50	1.75	0.75	0.00	1.25	**94.99**	0.00	0.00	0.00
**8**	0.00	0.00	0.00	0.00	0.00	1.18	0.00	**86.22**	0.00	12.06
**9**	0.00	0.00	0.00	0.00	0.00	0.78	0.78	0.00	**96.88**	1.56
**10**	0.00	0.37	0.00	0.37	0.00	1.49	0.00	4.48	0.00	**93.28**

**Table 2.** Non-crack feature detection results.

**Non-crack features**	**#samples**	**# of true positives**	**# of false positives**	**Recall (%)**	**Precision (%)**
Joints	278	234	25	84.17	90.34
White painting	1,764	1,679	2	95.18	99.88
Sealed cracks	173	166	9	95.95	94.85
**TOTAL**	**2,215**	**2,079**	**36**	**93.86**	**98.29**
